# Genome-Wide Association Study of Partial Resistance to *P. sojae* in Wild Soybeans from Heilongjiang Province, China

**DOI:** 10.3390/cimb44070221

**Published:** 2022-07-17

**Authors:** Wei Li, Miao Liu, Yong-Cai Lai, Jian-Xin Liu, Chao Fan, Guang Yang, Ling Wang, Wen-Wei Liang, Shu-Feng Di, De-Yue Yu, Ying-Dong Bi

**Affiliations:** 1 Crop Tillage and Cultivation Institute of Heilongjiang Academy of Agricultural Sciences (HAAS), Harbin 150086, China; nuio-3@163.com (W.L.); liumiao8349@163.com (M.L.); yame0451@163.com (Y.-C.L.); wendyliujx@163.com (J.-X.L.); beyean@163.com (C.F.); ouyanghuiru@163.com (G.Y.); lingling6958@163.com (L.W.); liangwenwei5@163.com (W.-W.L.); dishufeng2021@163.com (S.-F.D.); 2 College of Agriculture, Nanjing Agricultural University, Nanjing 210095, China; dyyu@njau.edu.cn

**Keywords:** genome-wide association study (GWAS), *Phytophthora sojae*, wild soybean, *Glycine soja*

## Abstract

Phytophthora root rot (PRR) is a destructive disease of soybeans (*Glycine max* (L.) Merr) caused by *Phytophthora sojae* (*P. sojae*). The most effective way to prevent the disease is growing resistant or tolerant varieties. Partial resistance provides a more durable resistance against the pathogen compared to complete resistance. Wild soybean (*Glycine soja* Sieb. & Zucc.) seems to be an extraordinarily important gene pool for soybean improvement due to its high level of genetic variation. In this study, 242 wild soybean germplasms originating from different regions of Heilongjiang province were used to identify resistance genes to *P. sojae* race 1 using a genome-wide association study (GWAS). A total of nine significant SNPs were detected, repeatedly associated with *P. sojae* resistance and located on chromosomes 1, 10, 12, 15, 17, 19 and 20. Among them, seven favorable allelic variations associated with *P. sojae* resistance were evaluated by a t-test. Eight candidate genes were predicted to explore the mechanistic hypotheses of partial resistance, including *Glysoja.19G051583*, which encodes an LRR receptor-like serine/threonine protein kinase protein, *Glysoja.19G051581*, which encodes a receptor-like cytosolic serine/threonine protein kinase protein. These findings will provide additional insights into the genetic architecture of *P. sojae* resistance in a large sample of wild soybeans and *P. sojae*-resistant breeding through marker-assisted selection.

## 1. Introduction

Phytophthora root rot (PRR), caused by the *Phytophthora sojae* pathogen, is one of the most destructive diseases of soybeans in world [1]. In China, PRR was first detected in Heilongjiang province in 1989. Subsequently, PRR spread to most soybean-producing areas, which caused significant yield losses each year [2,3]. Currently, the most effective ways to control PRR is to grow soybean cultivars, which confer resistance genes to *P. sojae* [4].

Two types of resistance to PRR have been reported in soybeans, including partial resistance, which is controlled by multiple genes, and complete resistance, which is mediated by the single dominant *Rps* resistance gene [5]. The management of PRR has primarily relied on single dominant resistance genes. A large amount of research on resistance-gene mapping has been conducted since the first resistance gene to *P. sojae* was identified in the 1950s [6]. To date, more than 33 *Rps* genes/alleles on 9 different soybean linkage groups/chromosomes have been identified and mapped: *Rps1a*, *Rps1b*, *Rps1c*, *Rps1d*, *Rps1k*, *RpsYu25*, and *Rps7* were in linkage group N; *Rps2* was in group J; *Rps3a*, *Rps3b*, *Rps3c*, and *Rps8* were in group F; *Rps4*, *Rps5*, and *Rps6* were in group G; and Rps1Su was in group O. Moreover, *Rps12*, *RpsHN*, *RpsQ*, *RpsGZ*, *Rps14*, and *Rps11* were reported as resistance genes to *P. sojae* [7,8,9,10,11,12,13]. *The Rps11* gene was located from Pi 594527 in chromosome 7 by using SNP genotyping [14]. Recently, a Phytophthora resistance gene *RpsWy* was mapped on chromosome 3 by high-throughput genome-wide sequencing [15]. Two candidate genes, *Glyma.03G033700* and *Glyma.03G033800*, conferring PRR against race 1 were also identified on chromosome 3 using the Specific Locus Amplified Fragment Sequencing (SLAF-seq) approach [16]. A novel Phytophthora resistance gene, *RpsZS18*, was detected on chromosome 2 of the soybean cultivar Zaoshu18 [17]. *RpsYD25* was predicted as a candidate gene and validated to be a diagnostic marker for *P. sojae* resistance breeding [18].

Complete resistance and partial resistance were not completely independent, the varieties with a complete resistance gene also had higher partial resistance levels. Partial resistance, known as horizontal resistance, was a quantitative trait controlled by QTL. Partial resistance could limit the spread of *P. sojae* in plant tissues and reduce the degree of root rot [19]. Recently, more than 70 quantitative trait loci (QTL) related to soybean partial resistance against *P. sojae* have been identified by genome-wide association studies (GWAS) [20]. *Glyma.13g32980*, *Glyma.13g33900*, and *Glyma.13g33512* were identified on chromosome 13 by GWAS based on naturally occurring variations of 279 accessions from Yangtze–Huai soybean breeding germplasms [21]. A major QDRL (quantitative disease-resistance locus) on chromosome 18 (*QDRL-18*) was identified in PI 427105B and PI 427106, which represents a valuable resistance source for breeding programs [22]. Moreover, some genes, such as *Glyma.01g32800*, *Glyma.01g32855*, and *Glyma.14g087500*, which are likely involved in PRR resistance, were identified. These works lay a foundation for exploring the mechanism of *P. sojae* resistance [23,24,25]. However, even though the rapid shift in quantities of *P. sojae* limits the effectiveness of resistance genes, the durability of an *Rps* gene is generally only 8–15 years [3,26]. Therefore, researchers must continuously search for more valuable resistance sources to identify new resistance genes.

Wild genetic resources play a significant role in transferring traits of interest, such as disease and insect resistance, improved quality, abiotic stress tolerance, and manipulation in modes of reproduction [27,28]. China is the origin and diversification center of the wild soybean that possesses many agronomically beneficial traits, such as high protein and lipid contents, adaptation to harsh conditions, and resistance to insects and disease [29]. Serving as valuable genetic resources, wild soybean harbors a high level of genetic variation and is certainly an extraordinarily important gene pool for soybean improvement [30]. However, many soybean collections, but few wild soybeans, were screened for exploiting novel resistance or tolerance sources [31,32,33].

Hence, the objectives of this study are to (i) detect the genetic resource presenting resistance and possibly carrying candidate genes or alleles by screening 242 wild soybeans from different regions in Heilongjiang province, (ii) map the resistance gene through a genome-wide association study (GWAS), and (iii) identify the candidate genes and their functional markers for marker-assisted selection.

## 2. Materials and Methods

### 2.1. Plant Materials

The resistance evaluation and correlation analysis of 242 soybean germplasms were conducted. These resources were obtained from 13 different ecological regions in Heilongjiang province and sampled according to the principle of representative and balanced sampling. All the materials were self-bred.

### 2.2. Medium and P. sojae Strain Preparation

Preparation of medium and strains are shown as follows:

Formula for medium: carrots (200 g) were juiced, boiled (30 min), and filtered; the volume was fixed to 1000 mL; and then agar (20 g) was added. This was then sterilized at 120 °C for 20 min. The prepared medium was poured into culture dishes with a thickness of 0.6 cm.

Culture of strain: race 1 of *P. sojae* was placed in the incubator at 25 °C for dark culture for 7 days.

### 2.3. Pot Experiment

Before planting, the soil was packed into small bags for sterilization at 121 °C for 1h. The seed coat was gently cut and broken with a knife, then sterilized with 75% alcohol for 50 s, and rinsed with sterile water 3–5 times. A total of 5 seeds from each material were planted in a pot and this was repeated 3 times. After emergence, 3 seedlings with consistent growths were kept in each pot. After the first three compound leaves were fully spread, inoculation and identification could be started. The materials were planted in batches every 7 days, which was repeated 3 times, After identification, fresh leaves were sampled and stored in a −80 °C refrigerator for DNA extraction. The CTAB method was used for DNA extraction [34].

### 2.4. Inoculation Identification

The leaf inoculation method was used in this study [35]. The three compound leaves that spread first were obtained and placed in a tray pre-arranged with sterile gauze. Distilled water was sprayed onto the bottom of the tray to keep the gauze moist. Cut a 0.5 cm wound in the center of each leaf with a blade. Then, cut the cultivated fungal medium into 0.5 × 0.5 cm pieces and inoculate it on the leaf wound with the growth side facing upward. The culture medium with no fungus was taken as the control. The inoculation test was repeated 3 times. The culture conditions were 24 °C, 12 h of light, and the relative humidity was 100%. After 5 days of inoculation, the disease status of the inoculated leaves was investigated. The standard of resistance identification is shown in Table 1 [35]. The formula for calculating the infection rate is as follows: infection rate = number of infected leaves (yellowing, browning, or yellowing)/total number of inoculated leaves × 100%.

### 2.5. Genotype Data and Quality Control

Data analysis was performed via R software (3.6.1 the 1me4 packages were loaded). The best linear unbiased prediction (BLUP) was obtained from the three-batch resistance to *P. sojae* of 242 accessions. The BLUP was used as the phenotypic value for association analysis. The calculation of the broad sense heritability (*h*^2^) was obtained using the equation *h*^2^ *= δ_g_*^2^*/(δ_g_*^2^ *+ δ_e_*^2^*)*, the variance of genetic variation and residual was calculated by the covariance of the genetic kinship matrix between individuals. Significant differences were evaluated by using one-way ANOVA and Duncan’s test at *p* ≤ 0.05. Tukey’s honest significant difference tests were conducted.

Genomic DNA was extracted by random disruption, DNA fragments were recovered, cluster was prepared by splicing, and enrichment amplification and sequencing were performed on Hiseq4000. The sequence data were compared with reference genome sequence by BWA software (0.7.17). When the level of mapping rate was below 70%, elimination was performed. SNPs were identified by Samtools (1.10) and Genome Analysis Toolkit (GATK4.0) [36]. SNP markers were excluded with a missing rate of >50% and a minor allele frequency (MAF) < 0.05.

### 2.6. Population Structure and Linkage Disequilibrium Analysis

The population heterozygosity (He), polymorphism information (PIC), and genetic diversity (π, θ) were calculated by VCF tools. The neighbor-joining tree was constructed using Phylip(3.5c). Population structure was calculated by fast structure [37]. Principal component analysis (PCA), which determines the population structure of *G. soja* accessions, was calculated using the R software package. The number of subgroups can be estimated by calculating the marginal likelihood. The pairwise linkage disequilibrium (LD) between SNP markers was calculated by using squared allele frequency correlations (r^2^) with PopLD decay.

### 2.7. Genome-Wide Association Analysis

The association analysis was performed with a general linear model (GLM) in GAPIT (3.0) [38]. The population structure was explained by PCA and the kinship was calculated by the Vanraden method [39].

### 2.8. Fluorescence Quantitative PCR Detection

The resistant and susceptible *G. soja* accessions were selected to screen differential expressions of candidate genes by fluorescence quantitative PCR. Samples were obtained from the stem 0.5 cm above and below the hypocotyl inoculation site at 0 h, 6 h, 12 h, 24 h, 36 h, and 48 h after inoculation, and were immediately frozen in liquid nitrogen and stored at −80 °C. RNA isolation was performed on each sample using a plant RNA Extraction Kit (Tiangen). The first cDNA strand was synthesized using the transcript RT Kit (Tiangen), according to the manufacturer’s instructions. Real-time fluorescent quantitative PCR was performed on a LightCycler480 II (Roche, Rotkreuz, Switzerland). Primer sequences of the candidate gene *Glysoja.19G051583* (F: CACCACCAAATCCCAGTT; R: AAGCACCAAAGACCAACAAAA), *Glysoja. 15 g042014* (F: AAAAGTTGCTGACCCATTGGTAAAT; R: TACCATACTGATGCTTACACGCT) were used for fluorescence amplification. The relative levels of transcript abundance were calculated using the underlying comparative threshold method 2^−(∆ Ct)^ with GmActin4 (GenBank accession no. AF049106) as the internal standard.

## 3. Results

### 3.1. Variation in Resistant Levels among G. soja Accessions

A total of 242 *G. soja* accessions originating from Heilongjiang province were evaluated for their response to one virulent isolate of *P. sojae*, race 1, using the leaf inoculation method. The susceptible rate for the inoculated accessions ranged from the lowest at 3.70% to the highest at 91.36%. The result’s phenotypic evaluation revealed a broad range of *P. sojae* resistance among the screened accessions. A total of 47 susceptible accessions, 167 intermediate accessions, and 27 resistant accessions were identified according to the standard of resistance; the percentage of genotyping was 19.50%, 69.29%, and 11.20%, respectively, among the tested accessions (Table 2).

**Figure 1:**
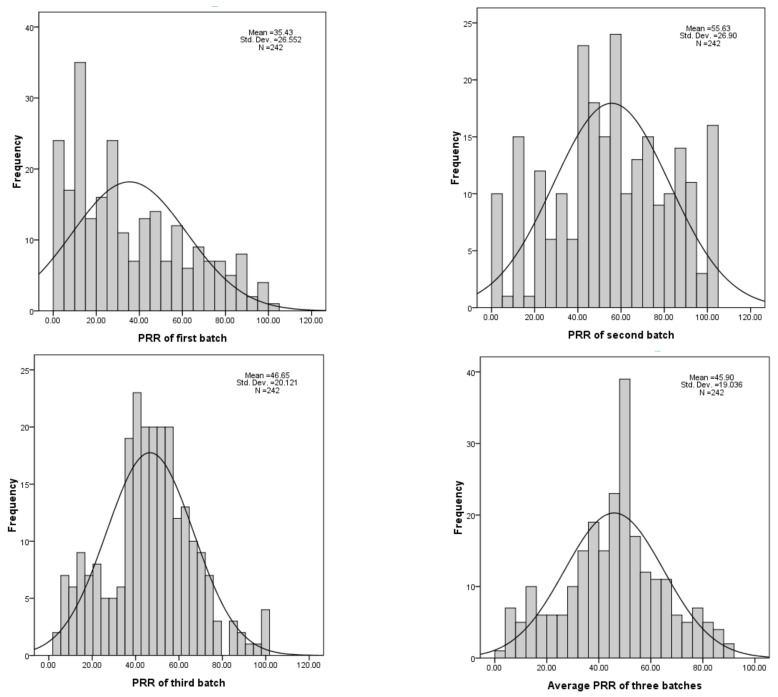
Frequency of *G. soja* resistance to *P. sojae*.

The phenotypic variations of three batches of *P. sojae* resistance were analyzed, including descriptive statistics, significance analysis, and generalized heritability (Table 3). The results show that there is a wide variation of resistance to *P. sojae* in the population, and the distribution is continuous (Figure 1). The kurtosis and skew of the first and third batches were all greater than 0, and the kurtosis and skew of the second batches were less than 0.

### 3.2. SNP Data

A total of 2.27 TB of data was acquired by high-throughput sequencing. According to the variation of SNPs, we calculated that the population’s He was 0.2898, PIC was 0.2389, diversity π was 1.45 × 10^−3^, and theta was 0.1576. A total of 4,152,769 SNPs were polymorphic in our data set (1 SNP/228 bp); a minimum minor allele frequency (MAF) of ≥5% was employed. Of the polymorphic SNPs, 999,800 had an MAF ≥ 5% and missing rate ≤ 50%, and these were evaluated in the present study for associations with *P. sojae* resistance.

### 3.3. Population Structure

**Figure 2:**
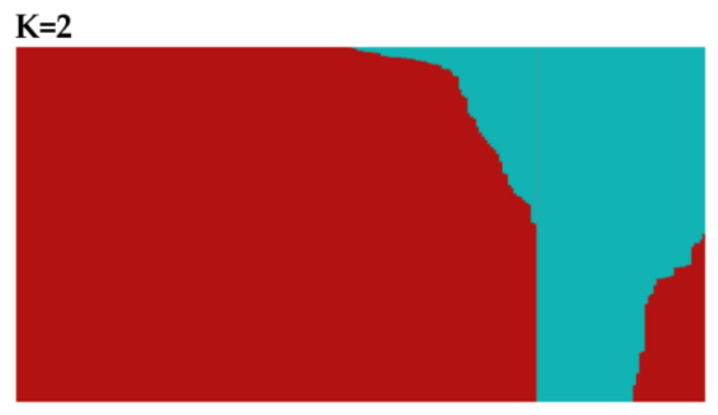
Population structure analysis of *G. soja*.

**Figure 3:**
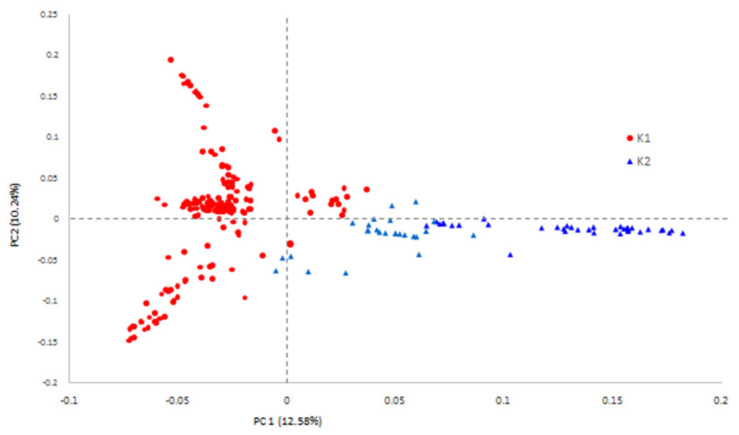
The PCA of *G. soja*.

**Figure 4:**
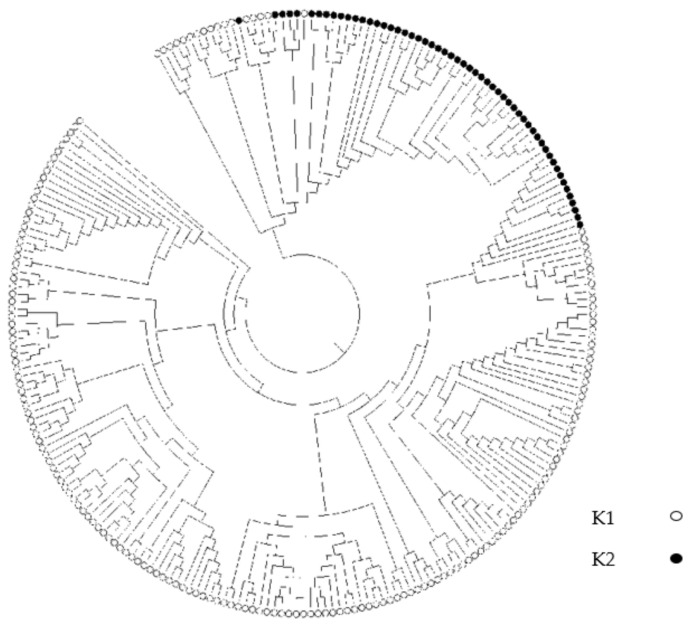
A neighbor-joining tree of *G. soja*.

The structure and relevance of soybean populations were analyzed. The marginal likelihood of population composition was estimated from 2 to 9 subgroups in turn. The 242 accessions were divided into two sub-populations (clusters), K1 (red) and K2 (blue), based on structure analysis, as the maximal delta K value was observed when K = 2 (Figure 2). Sub-population K1 represented typical wild soybean accessions with smaller seeds, and sub-population K2 was predominantly composed of semi-wild soybeans with larger seeds. From the principal component analysis (PCA) (Figure 3), the total amounts of genetic variations explained by the first two principal components were 12.58% and 10.24%. The first two principal components visually differentiated accessions into wild soybeans and semi-wild soybeans, which were consistent with the structure analysis. Sub-populations K1 and K2 were clustered together in the phylogenetic tree analysis (Figure 4), respectively, which displayed consistent results in agreement with the population structure analysis. 

### 3.4. Linkage Disequilibrium

**Figure 5:**
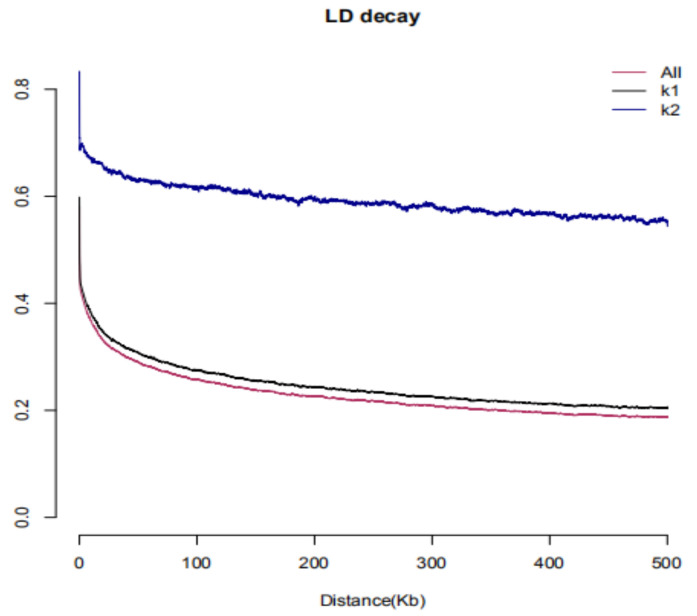
Analysis of LD.

The linkage disequilibrium (LD) was calculated using 999,800 SNPs with a minor allele frequency ≥ 5% covering the 20 chromosomes. LD decayed to an r^2^ of 0.2 at approximately 50 kb for the whole population. While the LD value of subgroup 1 was 60 kb, the decrement distance of subgroup 2 could not be obtained (Figure 5).

### 3.5. Genome-Wide Association Analysis

**Figure 6:**
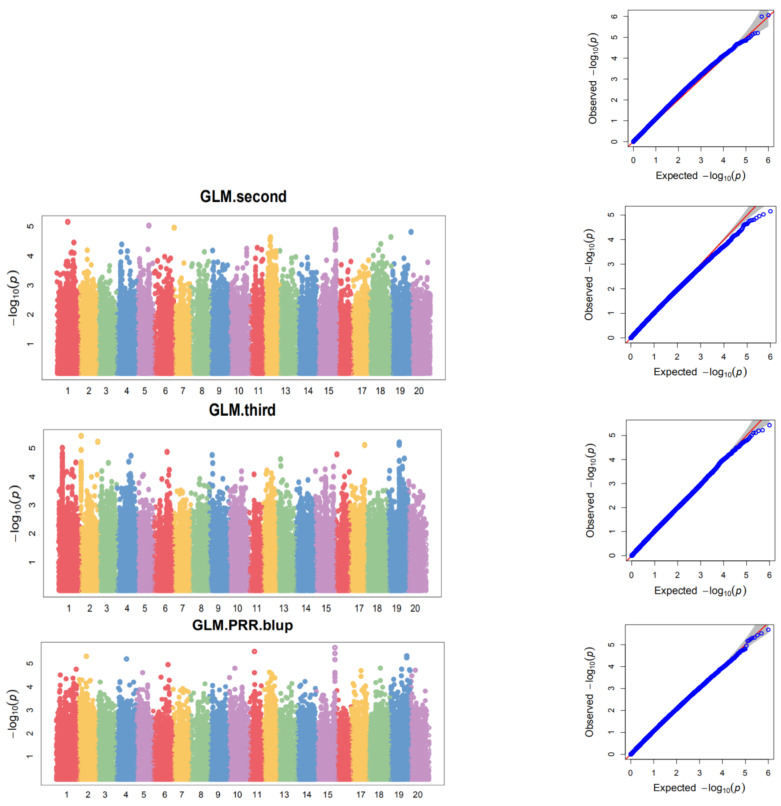
Manhattan and QQ plots of GWAS for wild soybeans resistance to PRR.

A total of 79 SNPs were identified to be significantly associated with resistance; at least one batch tested *P. sojae* race 1 at the level of −log_10_ (*p*) ≥ 4.5 in the GLM analysis (Figure 6, Table 4). We identified 14 SNPs associated with race 1 for the first batch, 15 SNPs were associated with race 1 for the second batch, and 25 SNPs were associated with race 1 for the third batch. Moreover, 25 SNPs were identified to be associated with race 1 for the BLUP. To evaluate the potential resistance gene of *P. sojae*, the methods used in this study applied several approaches to avoid *Rps*-mediated responses. A total of 9 SNPs were found to be repeatedly associated with race 1 for both the batch and the BLUP, and were located on chromosomes 1, 10, 12, 15, 17, 19, and 20 (three sites on chromosome 19). The phenotypic variation explanation of 9 association SNPs ranged from 7.43% to 10.05% (Table 5).

To confirm the reliability of resistance-associated markers identified by the GLM method, as shown in Table 6, for each variation, the accessions were divided into two groups based on the variations of SNPs. A *t*-test was performed for the mean value of the susceptible rate between the two groups. The susceptible rate of accessions with alleles that were significantly lower in disease resistance can be selected as reliable variants for screening favorable germplasm resources; meanwhile, alleles with no significant differences in disease resistance were unreliable. A total of seven favorable allelic variations (rs10641-T, rs532502-T, rs718743-C, rs922217-G, rs938638-G, rs940996-C, and rs1958957-T) were detected in nine significant associated alleles (Table 6). The typical carrier accessions were HAAS_077 and HAAS_264.

### 3.6. Prediction of Candidate Genes for PRR Resistance

As the stable resistance-associated SNPs located on different chromosomes were consistently identified to be associated with the *P. sojae* resistance in all batches, we performed the candidate gene prediction analysis in the genomic region surrounding the even associated SNPs (Table 6). According to the LD distance, we extended and selected the region of 500 kb upstream and downstream of the peak SNP marker on both sides. We found that four SNPs were located within the gene, the other three SNPs were located in intergenic regions. A total of 30 candidate genes were predicted within the search region (Table 7). Based on the detailed annotations for soybean reference genomes in SoyBase (http://www.soybase.org, accessed on 8 April 2020), or wild soybean candidate genes in NCBI (http://www.ncbi.nlm.nih.gov, accessed on 8 April 2020), 8 candidate genes were predicted from these 30 genes for possibly regulating *P. sojae* resistance in soybeans and considered to be candidate genes associated with PRR resistance (Table 8). This candidate list included genes encoding resistance to Phytophthora-related proteins, receptor-like kinase proteins, a caffeoyl-CoA O-methyl transferase, and glutathione S-transferase. Two identified *RKF3* genes (*Glysoja.19G051583* and *Glysoja.19G051582*) and one *RBK2* gene (*Glysoja.19G051581*) were close to SNP rs938638. The gene *Glysoja.19G051582* was only 1 kb away from SNP rs938638, the gene *Glysoja.19G051581* was 4 kb away from it, and the gene *Glysoja.19G051583* was 7.6 kb away from SNP rs938638.

### 3.7. Expression of Candidate Gene’s Response to P. sojae Infection in Resistant and Susceptible Germplasms

**Figure 7:**
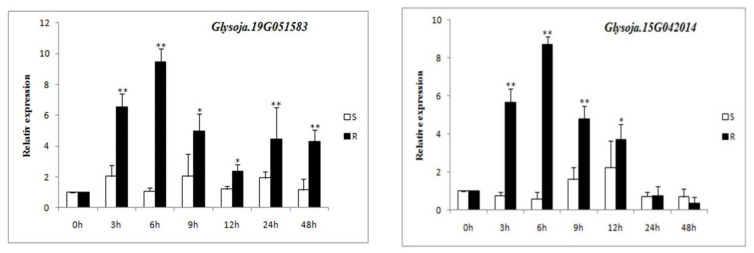
Relative expressions of candidate genes were induced by *P. sojae*. Note *: 0.05 level; **: 0.01 level.

Quantitative real-time reverse transcription PCRs (qRTPCRs) showed that expressions of *Glysoja.19G051583* and *Glysoja.15G042014* were obviously induced by *P. sojae* infection in the resistant wild soybeans. The abundance of gene expression varied along the processing times at 0 h, 3 h, 6 h, 9 h, 12 h, 24 h, and 48 h after inoculation with Phytophthora infestans. However, the gene expression at 6 h after inoculation was highest for both *Glysoja.19G051583* and *Glysoja.15G042014*, followed by 9 h and 3 h. Importantly, the gene expression was significantly (*p* < 0.01) higher in resistant germplasms in comparison to susceptible germplasms (Figure 7). The gene *Glysoja.19G051583* was up-regulated within 48 h after inoculation, compared to susceptible germplasms, while the *Glysoja.15G042014* gene was up-regulated within 12 h after inoculation, compared to susceptible germplasms.

## 4. Discussion

PRR is one of the most serious diseases in soybeans and has caused a great reduction in soybean production in recent years. The application of resistant varieties seemed to be the most effective way to control PRR. However, the widespread use of complete resistance genes can lead to the adaptation of *P. sojae* populations to the deployed resistance. Searching for more valuable resistance sources has become important to develop cultivars with increased levels of partial resistance. A large quantity of soybean germplasms have been screened for PRR resistance [31,32,33,40,41]. Wild soybean is an extraordinarily important gene pool for soybean breeding. In this study, 27 resistant wild soybeans were identified in response to race 1 of *P. sojae*, the dominant race of PRR; these works could be useful for breeding and the genetic research on resistance to *P. sojae*. The intention of this investigation was to identify SNPs by GWAS and candidate genes that play an important role in the PRR resistance variation in our wild soybean population.

For the GWAS analysis, we used the GLM method to identify the markers associated with PRR resistance. By using the genotypic data of 999,800 SNPs with MAF ≥ 5%, a total of 79 SNPs were identified to be significantly associated with the resistance to *P. sojae* race 1 of at least one tested batch at the level of −log10 (*p*) ≥ 4.5. Among these SNPs, 9 SNPs were detected to be associated with race 1 for both the batch and BLUP. Compared to previous GWAS studies on *P. sojae* resistance, our resistance-associated regions were either not on the same chromosomes or were at various distances from the reported alleles and QTLs. Niu et al. (2018) used 337 accessions to identify resistance regions associated with PRR resistance by GWAS, 26 significant SNPs associated with Phytophthora resistance were detected on chromosome 1, and no previous studies have reported resistance loci in this 441 kb region [23]. Schneider et al. (2016) used 1395 Korea accessions to identify seven QTLs on Chr. 3, 13, and 19 associated with partial resistance to *P. sojae* [42]. The SNPs we identified for race 1 were on Chr. 1, 10, 12, 15, 17, 19, and 20, while Qin et al. (2017) identified six SNPs located on Chr. 3, 5, 13, and 18 associated with race 1. The result show that ss715614943 on Chr. 13 has the highest significant association with *P. sojae* race 1 with an LOD value of 4.46 in the GLM analysis [24]. Li et al. (2016) also identified a resistance-associated region containing three candidate genes (*Glyma.13g32980*, *Glyma.13g33900*, and *Glyma.13g33512*) on chromosome 13 [21]. The highest significant association rs718743 was identified on Chr. 15 with an LOD value of 5.68 in the GLM analysis in our result. Interestingly, a relatively major effect *P. sojae* resistance QTL was identified on Chr. 15 through whole-genome resequencing using a diverse panel of 357 soybean accessions in the previous study [20]. Moreover, in our study, a total of seven favorable allelic variations (rs10641-T, rs532502-T, rs718743-C, rs922217-G, rs938638-G, rs940996-C, and rs1958957-T) were identified to be candidate regions for resistance to *P. sojae* in wild soybeans on chromosomes 1, 12, 15, 19, and 20, which had not been reported. It indicated that these seven favorable materials, including HAAS_077, which carries six tightly resistant associated alleles, could be useful for germplasm innovation and molecular marker-assisted breeding. For candidate gene identification, we discovered even significant SNPs on five different chromosomes that were associated with *P. sojae* resistance in our wild soybean sample. SNPs in clusters, especially those on chromosomes 15 and 19, are probably the most interesting and worth further investigation. Eight candidate genes involved in plant defense-related reactions were identified here. *Glysoja.15G042020* and *Glysoja.15G042019*, which encode Glutathione S-transferase, were detected in the 48099201–48129356 region on chromosomes 15. A number of studies have reported that Glutathione S-transferase, caffeoyl-CoA O-methyltransferase, LRR receptor-like serine/threonine protein kinase, and receptor-like protein kinases play important roles in plant defense-related reactions against fungal attacks [43,44,45,46,47,48]. Jing et al. (2015) found that the expression of the GST family in soybeans was down-regulated following *P. sojae* infections [49]. These results imply that *Glysoja.15G042020* or *Glysoja.15G042019* are likely candidate genes conferring resistance to *P. sojae*. Receptor-like protein kinases (RLKs) and other stress-related plant protein kinases have been found to be involved in signal transduction. The RLKs located on plant cell membranes have attracted considerable attention in the study of plant signal pathways [50,51]. Interestingly, three of the candidate genes (*Glysoja.19G051582*, *Glysoja.19G051583*) were identified close to SNP rs938638, which encode LRR receptor-like serine/threonine protein kinase located on chromosome 19 in the region of 41190378-41199245. Four serine/threonine protein kinase-coding genes are mapped and annotated in the region that is a well-known location for *Rps1* and *Rps7* in a previous study [52]. Furthermore, the expression of *Glysoja.19G051583* reached the highest level at 6 h after inoculation. Importantly, the gene expression was 10-fold greater in resistant germplasms compared with susceptible germplasms. Li et al. (2017) found that *Glyma.03g27200* encoding a protein with a typical serine/threonine protein kinase structure and the expression pattern analysis showed that this gene was induced by *P. sojae* infection, which was suggested as the best candidate gene for *RpsQ* [9]. Further research revealed that *RpsX* and *RpsQ* share common nonsynonymous SNPs and a 144-bp insertion in the *Glyma.03g027200* sequence encoding a leucine-rich repeat (LRR) region, which may be important for PRR resistance in soybeans [3]. The results of the present study provide foundational knowledge for researchers who are interested in soybean–*P. sojae* interactions. A further characterization should focus on validating the role of candidate genes against *P. sojae* and modulating the resistance between the accessions carrying the R or S alleles in this population.

## 5. Conclusions

In the present study, a GWAS was performed to detect genomic regions contributing to partial resistance to *P. sojae* using wild soybean accessions obtained from Heilongjiang province China. Nine SNPs were detected to be repeatedly associated with race 1 and were located on chromosomes 1, 10, 12, 15, 17, 19, and 20. Some SNPs that coincided with previously reported QTLs for resistance to *P. sojae* were identified. A total of eight candidate genes were predicted to explore mechanistic hypotheses of partial resistance, including *RKF3* and *RBK2*, which was involved in morphology and development, basal defense, and signal transduction. Some of these SNPs may be useful for *P. sojae* resistance breeding. Our results also provide additional insights into the genetic architecture of *P. sojae* resistance in a large sample of wild soybeans.

## Figures and Tables

**Table 1:** Standard of resistance to *P. sojae*.

Reaction	Standard of Identification	Susceptible Rate (%)
R	Yellowing, browning, or chlorosis of leaves	<30
I		30–70
S		>70

R: resistance; S: susceptible; I: intermediate.

**Table 2:** Analysis of resistance identification for *G. soja* accessions.

Reaction	Numbers	Percent
S	47	19.50
I	167	69.29
R	27	11.20

**Table 3:** Descriptive statistics of *G. soja* resistance to *P. sojae*.

Batch	Skew	Kurtosis	Heritability
First	0.62	−0.67	65.82
Second	−0.17	−0.73	
Third	0.08	0.07	

**Table 4:** SNPs associated with *G. soja* resistance to *P. sojae*.

Batch	SNP	Chr	Position	SNP	Chr	Position
First	rs64989	1	51,714,188	rs494319	11	15,826,009
	rs70530	2	9,109,207	rs494372	11	15,827,702
	rs70550	2	9,110,030	rs497213	11	17,412,064
	rs70646	2	9,120,078	rs498507	11	17,862,252
	rs264115	6	21,032,877	rs570096	13	1,285,457
	rs434454	10	8,904,675	rs779074	17	24,607,598
	rs444818	10	14,862,256	rs885444	19	8,001,494
Second	rs32673	1	27,448,011	rs717476	15	46,519,729
	rs248180	5	30,006,501	rs717478	15	46,519,754
	rs299633	7	625,039	rs717479	15	46,519,777
	rs530881	12	15,479,974	rs717480	15	46,519,780
	rs530911	12	15,481,110	rs718743	15	47,944,309
	rs532502	12	16,557,015	rs875366	18	56,051,504
	rs717197	15	46,275,173	rs940996	19	49,218,250
	rs717475	15	46,519,716			
Third	rs9312	1	10,161,448	rs193415	4	30,457,058
	rs9748	1	10,439,969	rs200863	4	36,132,462
	rs10026	1	10,532,649	rs281244	6	34,683,089
	rs10216	1	10,586,571	rs371411	9	6,767,103
	rs10270	1	10,593,132	rs576319	13	6,313,762
	rs10380	1	10,641,555	rs721959	16	5,055,148
	rs10436	1	10,669,494	rs801645	17	38,595,915
	rs10641	1	10,725,370	rs921647	19	27,986,596
	rs67599	2	2,186,517	rs921800	19	28,122,057
	rs67717	2	2,190,064	rs921801	19	28,122,124
	rs67718	2	2,190,067	rs922217	19	28,376,899
	rs67720	2	2,190,157	rs938638	19	41,267,399
	rs116637	2	45,054,777			
BLUP	rs10641	1	10,725,370	rs718675	15	47,928,197
	rs64791	1	51,140,109	rs718693	15	47,938,751
	rs85323	2	20,208,766	rs718743	15	47,944,309
	rs190201	4	27,955,786	rs718756	15	47,944,902
	rs230678	5	16,483,623	rs779074	17	24,607,598
	rs286790	6	38,875,969	rs844904	18	32,247,141
	rs444818	10	14,862,256	rs922217	19	28,376,899
	rs490370	11	12,974,021	rs938637	19	41,267,263
	rs490521	11	13,035,152	rs938638	19	41,267,399
	rs532502	12	16,557,015	rs940814	19	48,382,478
	rs539755	12	21,078,905	rs940996	19	49,218,250
	rs545454	12	22,657,211	rs958957	20	12,404,540
	rs718653	15	47,921,059			

**Table 5:** The SNPs repeatedly associated with PRR.

Chr	SNP	Batch	Position	*p* Value	−log_10_(*p*)	Maf	Phenotypic Variation (%)
1	rs10641	Third	10,725,370	1.98 × 10^−5^	4.70	0.13	8.21
		BLUP		3.13 × 10^−5^	4.50		7.43
10	rs444818	First	14,862,256	1.46 × 10^−5^	4.84	0.12	7.75
		BLUP		1.60 × 10^−5^	4.80		8
12	rs532502	Second	16,557,015	2.35 × 10^−5^	4.63	0.33	7.63
		BLUP		2.37 × 10^−5^	4.63		7.67
15	rs718743	Second	47,944,309	2.36 × 10^−5^	4.63	0.30	7.63
		BLUP		2.07 × 10^−6^	5.68		9.77
17	rs779074	First	24,607,598	1.27 × 10^−5^	4.90	0.17	7.59
		BLUP		2.03 × 10^−5^	4.69		7.8
19	rs922217	Third	28,376,899	2.79 × 10^−5^	4.55	0.19	7.91
		BLUP		1.75 × 10^−5^	4.76		7.92
	rs938638	Third	41,267,399	2.33 × 10^−5^	4.63	0.36	8.07
		BLUP		4.71 × 10^−6^	5.33		9.05
	rs940996	Second	49,218,250	1.55 × 10^−5^	4.81	0.42	7.98
		BLUP		2.00 × 10^−5^	4.70		7.81
20	rs958957	First	12,404,540	5.29 × 10^−6^	5.28	0.41	10.05
		BLUP		1.95 × 10^−5^	4.71		7.83

**Table 6:** Favorable allele effects and carrier accessions.

SNP	Chr	Position	Allele	Mean of Susceptible Rate	Allele	Mean of Susceptible Rate	*t*-Test	Significant Material
rs10641	Chr01	10,725,370	C	48.45	T	37.04	0.0038	HAAS_077
rs532502	Chr12	16,557,015	C	59.4	T	48.78	0.0034	HAAS_264
rs718743	Chr15	47,944,309	A	63.98	C	51.14	0.0010	HAAS_264
rs922217	Chr19	28,376,899	A	54.22	G	44.49	0.0089	HAAS_077
rs938638		41,267,399	T	52.31	G	43.92	0.0018	HAAS_077
rs940996		49,218,250	C	47.83	G	60.20	0.0002	HAAS_077
rs958957	Chr20	12,404,540	C	48.44	T	37.96	0.0003	HAAS_077

**Table 7:** Prediction of candidate genes.

SNP	GENE ID	Annotations
Chr15 rs718743	***Glysoja.15G042021***	**Putative glutathione S-transferase parC**
	***Glysoja.15G042020***	**Putative glutathione S-transferase**
	***Glysoja.15G042019***	**Putative glutathione S-transferase**
	*Glysoja.15G042017*	Nicotianamine synthase
	*Glysoja.15G042016*	Ubiquitin carboxyl-terminal hydrolase 25
	*Glysoja.15G042015*	Putative sugar phosphate/phosphate translocator
	***Glysoja.15G042014***	**Putative caffeoyl-CoA O-methyltransferase 1**
	*Glysoja.15G042012*	(S)-2-hydroxy-acid oxidase GLO1(s)
Chr19 rs922217	*Glysoja.19G050845*	Elongation factor 1-alpha
Chr19 rs938638	***Glysoja.19G051587***	**Protein resistance to Phytophthora 1, chloroplastic-like**
	*Glysoja.19G051585*	Sugar transporter ERD6-like 7
	***Glysoja.19G051583***	**Putative LRR receptor-like serine/threonine protein kinase RKF3**
	***Glysoja.19G051582***	**Putative LRR receptor-like serine/threonine protein kinase RKF3**
	***Glysoja.19G051581***	**Receptor-like cytosolic serine/threonine protein kinase RBK2**
	*Glysoja.19G051580*	Autophagy-related protein 18 g
	*Glysoja.19G051579*	Histidine-containing phosphotransfer protein AHP1
	*Glysoja.19G051577*	Pentatricopeptide repeat-containing protein
	*Glysoja.19G051576*	Gibberellin receptor GID1B
	*Glysoja.19G051575*	Hypothetical protein
Chr19 rs940996	*Glysoja.19G052510*	Receptor-like protein kinase ANXUR2
	*Glysoja.19G052507*	Pathogenesis-related protein PR-4A
	*Glysoja.19G052505*	Pro-hevein
	*Glysoja.19G052504*	Auxin-responsive protein IAA16-like
	*Glysoja.19G052503*	Mediator of RNA polymerase II transcription subunit 14RNA
	*Glysoja.19G052502*	Sec-independent protein translocase protein TATA, chloroplastic
	*Glysoja.19G052501*	Light-inducible protein CPRF2
	*Glysoja.19G052500*	GDP-mannose 3,5-epimerase 1
	*Glysoja.19G052499*	GDP-mannose 3,5-epimerase 1
	*Glysoja.19G052497*	Ammonium transporter 3 member 1
	*Glysoja.19G052496*	Calmodulin-like protein 8

**Table 8:** Candidate genes associated with PRR resistance.

GENE ID	GENE Name	Position	SNP	SNP Location
*Glysoja.15G042021*	*PARC*	48,129,405–48,131,670	rs718743	48,076,672
*Glysoja.15G042020*	*GST*	48,126,966–48,129,356		
*Glysoja.15G042019*	*GST*	48,099,201–48,101,217		
*Glysoja.15G042014*	*Omt5*	48,039,247–48,039,770		
*Glysoja.19G051587*	*PRR1*	41,224,586–41,226,602	rs938638	41,190,696
*Glysoja.19G051583*	*RKF3*	41,197,436–41,199,245		
*Glysoja.19G051582*	*RKF3*	41,190,378–41,192,993		
*Glysoja.19G051581*	*RBK2*	41,184,811–41,188,366		

## Data Availability

Not applicable.
